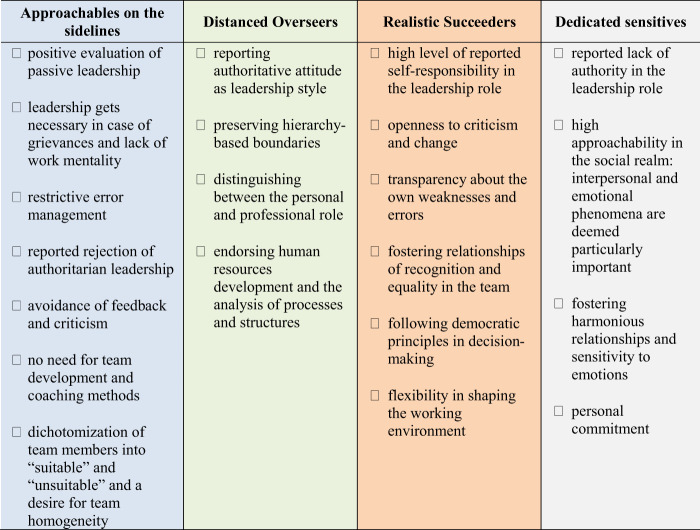# From approachables on the sidelines to dedicated sensitives: developing a leadership typology among healthcare leaders utilizing grounded theory

**DOI:** 10.1108/JHOM-01-2022-0018

**Published:** 2022-10-03

**Authors:** Janna Katharina Küllenberg, Debora Niermann, Sonja Becker, Mirjam Körner

**Affiliations:** Institute of Medical Psychology and Medical Sociology, University of Freiburg , Freiburg, Germany; Department of Occupational and Consumer Psychology, Institute of Psychology, University of Freiburg , Freiburg, Germany; Zurich University of Teacher Education , Zurich, Switzerland

**Keywords:** Coaching, Leadership, Rehabilitation, Grounded theory, Teamwork

## Abstract

**Purpose:**

Based on a resulting typing model, this paper focuses on four types of leaders (Approachables on the sidelines, Distanced overseers, Realistic succeeders and Dedicated sensitives), who differ in the analytical core category of “development of awareness.”

**Design/methodology/approach:**

Internal team coaching is intended to strengthen leaders in the health care system. The Team Leader Coaching Programme (TLCP) was implemented as an internal coaching instrument at rehabilitation centers using a train-the-trainer format. Twenty-one team leaders were surveyed on their experience of the coaching process they implemented in their teams. The interviews were analyzed using the grounded theory method (GTM) as theoretically discussed by representatives of second-generation GTM (Charmaz, 2014).

**Findings:**

Use of the TLCP proved to be an intervention for initiating and enhancing an awareness development process regarding team leaders' reflections on their own position and leadership role, regardless of their profession. This process was found to be a prerequisite for implementing the learned content. The typing model is discussed given current contextual conditions in the rehabilitation system and their connectivity in practice for integrating coaching elements into daily management.

**Originality/value:**

This article presents a typology of healthcare leaders. Thanks to the reconstructive approach using grounded theory methodology, this article presents an in-depth analysis of the implementation process of a coaching program. The findings are both connectable to applied leadership research and useable for further development of training and interventions to strengthen team leaders in clinical settings.

## Introduction

Ensuring optimal healthcare delivery and managing the complexity of patient care requires that different professions in health and social services collaborate effectively ( Côté *et al.* , 2008 ). In the healthcare sector, teams must increasingly generate new and innovative approaches to solve complex health-related problems ( Gilbert *et al.* , 2010 ). Complexities emerge, for instance, where the fundamental changes in healthcare organizations are accompanied by greater psychosocial challenges. They include financial constraints, managed care, consumer behavior, new information technologies, the changed role of physicians as advisors and information mediators ( Bartholomeyczik *et al.* , 2008 ) as well as time pressure in patient visits, which causes dissatisfaction in many areas ( Linzer *et al.* , 2000 ).

### Background

#### Interprofessional collaboration

Interprofessional collaboration (IPC) is a process in which various professions work toward a shared goal by making different but complementary contributions to patient-centered care and integrating it into comprehensive treatment ( Lingard *et al.* , 2012 ; Zwarenstein *et al.,* 2009 ). Particularly in the medical rehabilitation setting, IPC is viewed as a key criterion for quality and success ( Davis *et al.* , 1992 ; Müller *et al.,* 2014 ). It is essential not only for effective and efficient patient care, but also for the job satisfaction of care providers ( Körner *et al.* , 2015 ). In recent years, it was found that the synergistic effects of interprofessional teams are not fully exploited. This is partly due to the continuous promotion of professional specialization, which tends to lead to isolation of the professions, silo mentality and potential interprofessional communication problems and conflicts ( Gilbert *et al.* , 2010 ).

#### Leadership in the healthcare sector

Efficient leadership is essential for ensuring patient safety ( Gilardi *et al.* , 2014 ; Rosenman *et al.* , 2014 ; Sims *et al.,* 2015 ). It can maximize team potentials and ensure higher-quality care ( Weaver *et al.* , 2014 ). To ensure efficient leadership, leaders must establish a clear direction and provide support in supervision ( Nancarrow *et al.* , 2013 ). Specifically, leaders in healthcare are believed to require more extensive social skills and high emotional intelligence ( Henochowicz and Hetherington, 2006 ) because facilitating employees' personal development, listening and acting exemplary are important management tasks ( Nancarrow *et al.* , 2013 ). To utilize the potential of different professions, interdisciplinary teams can create a shared leadership role, with one person being responsible for managing, developing and coaching a team of professionals ( McCallin, 2003a ). In shared leadership, team members temporarily assume a leadership role depending on the needs of the environment and specific circumstances. Thus, leadership does not rest with the individual, but is shared among several people working toward a common goal ( Pearce *et al.* , 2011 ). The transfer of leadership tasks from leaders to team members should tap members' strengths, knowledge, skills, attitudes, perspectives, contacts and available time ( Burke *et al.* , 2003 ). An integrative approach for strengthening leaders in the healthcare sector is team coaching, used as a leadership tool ( Hackman and Wageman, 2005 ). The objective is to establish clear expectations to increase the members' identification with the desired results, provide recognition and feedback, encourage good performance and identify areas to be improved, stimulate problem solving as well as to motivate team members to break new ground in an effort to anticipate problems before they arise ( Dimas *et al.* , 2016 ). The Team Leader Coaching Programme (TLCP), an internal coaching and leadership tool is based on the “patient-oriented team development” approach ( Körner *et al.* , 2016 ), developed for the medical rehabilitation context ( Becker *et al.* , 2017 ). The goal of this study is to analyze subjective perspectives and experiences of internal team coaches after implementation of the training to generate contextualized knowledge.

#### Training development and implementation

The TLCP was implemented as an intervention in a train-the-trainer format in Küllenberg *et al.* (2021) . Team leaders received two days of training, consisting of two modules held eight weeks apart. Between the modules, participants had the opportunity to begin initial team development, with support being available (manual and telephone contact with the trainers). Module 1 included an introduction to various leadership styles, familiarization with and trying out coaching tools and systemic questions, exercises for critical self-reflection on one's leadership role, interventions for the development of a coaching attitude as well instructions for developing a vision for one's team. The contents of module 2 followed up on those of module 1, starting with positioning team leaders within their process, further moderation techniques, the solution finding method and securing of results ( Küllenberg *et al.* , 2021 ).

## Methods

### Design of the study

The analysis used a grounded theory approach: as an interpretive research program, it aims to generate knowledge about social processes. The underlying idea is that individuals within groups define themselves in situations inside and outside the group, resulting in the development of shared patterns of behaviors ( McCallin, 2003b ). The investigators participated in the research process as trainers. Utilizing empirical knowledge, an approach was chosen that considers the reflexivity of the researcher's role in the research process and incorporates it into the analysis. The researchers' perspective is based on constructivist epistemology in line with the theoretical analyses of representatives of second-generation GTM ( Charmaz, 2014 ). Methodologically, the study followed Strauss and Corbin (1996) and Charmaz (2006) .

### Ethics

The study was approved by the medical ethics committee of Freiburg University Hospital (number 96/17).

### Data collection

Telephone interviews were conducted with team leaders ( *n* = 21) from rehabilitation centers in Germany who implemented the TLCP in their teams. Inclusion criteria for participants in the interviews were a leadership role and completion of the entire two-day training. Professionally, participants were nurses, quality managers, occupational therapists, physical therapists, physicians and psychologists. The interviews lasted 42 min on average. Written declarations of consent were obtained before the interviews were conducted. Before starting any recordings, aspects relevant to data protection were pointed out and the start of the recording was signaled. The interviews were transcribed based on the extended rules of Dresing and Pehl (2010) and then pseudonymized by the research team. The interviews followed a semi-structured interview guide developed by a group of three researchers using the S2PS2 method (sammeln [collect], sortieren [sort], prüfen [check], streichen [eliminate], subsumieren [subsume]) ( Kruse, 2015 ). Structurally, the guide starts with an open entry question, aiming to elicit narrations: “Some months have passed since the first day of the workshop. I'm interested to learn what has happened at your center since that time. Tell me: How did you experience the past three months?” This entry question is supplemented by more detailed questions. The guide includes questions on attitudes toward the process and experiences of the coaching and leadership role, change factors, structure and culture on the team and organizational levels, as well as any changes that took place and final questions about the study. The guide was gradually developed in an iterative data collection and analysis process, beginning after the first ten interviews. The interviewers kept logs in which they recorded aspects related to the communication, interview atmosphere, environment (interruptions, pauses, or other distinctive features) and the interviewers' reflections on their perception of the interview. Two of the four interviewers were trainers of the program. As a team, we were concerned about whether this dual role might cause difficulties in the interviews. It was, therefore, essential for us to reflect on this situation. This is one of the reasons why we deliberately kept the questions in the interview situation open. Our observation was that our research participants felt that they could speak freely. In principle, the willingness to provide information and participate was high and we attribute this to the rapport established beforehand. However, because we also wanted to protect ourselves in the analysis process from hasty attributions, we took further measures for the data analysis (see below). Case vignettes were created for all interviews and structured using the following aspects: perceived interview atmosphere, implementation of coaching, team characteristics, team leader characteristics, suggested *in vivo* codes, topics addressed, linguistic and content-related peculiarities, thematization rues, attitude toward the workshop, desires, focus for sections, sections for detailed analysis.

### Setting

Data were analyzed in a research team consisting of four project team members, supported at regular intervals through supervision by an expert in grounded theory methodology. More precisely, two of the four participants in the analysis group were trainers of the program. To ensure openness and create more heterogeneity, the analysis group was expanded by two researchers who were not familiar with the participants. This group, in turn, was supervised by a fifth person with methodological expertise in GTM. To reduce categorization processes throughout the analysis process, the participant IDs and personal information were not salient but replaced with pseudonyms, so contrasting cases were not directly related to persons.

### Analysis

Sections for detailed analysis were selected by looking for passages generating maximum narrative; the approach for developing shared understanding was based on Charmaz (2014) . In the group, sensitizing concepts were made transparent and critiqued to be used as a starting point for theory building rather than perceptual closure ( Charmaz, 2006 ). Therefore, the trainers' contextual knowledge increased sensitivity in theory building and variability. The external expert instructed the two researchers who were not trainers in the clinics to be “advocates diaboli” and constantly pushed the contrasting sides. The composition of the analysis group had the intention that previous knowledge can strengthen a research project, when carefully navigating and controlling perspectives rather than attempting to erase or forget their knowledge or experiences ( Charmaz, 2014 ).

The coding process was carried out following Strauss and Corbin (1996) based on a three-stage, interlinked approach of open coding, axial coding and selective coding. To significantly aid structuring for theory generation, coding paradigms were followed in the context of axial coding ( Breuer *et al.* , 2017 ). Within the coding paradigm, codes were analyzed with regard to causes, context, strategies, intervening conditions and consequences and differentiated using constant comparative analysis techniques and following the principle of maximum, meaningful contrasts ( Corbin and Strauss, 2008 ).

### Findings

The subsequent typology represents specific leadership styles in practice. Four types of team leaders were identified who differ in the analytical core category of “development of awareness.” Awareness developed when team leaders started to reflect on their leadership role and simultaneously explicitly reported using the learned coaching contents. The reported changes in terms of reflecting on their leadership role (agency and positioning, perception of the self-concept and of the team, narrative self-reflective moments) on the one hand and the reported implementation process of coaching elements (reported experiences, making of space and time, resource use) on the other hand, led to positioning of the cases in the analytical core category. The results presented below all relate to the positioning of the team leaders within the core category of “development of awareness.” Positioning within the core category was determined using decision parameters in the form of central questions on the axes of the degree of reflection and commitment (see Table 1 ). However, any one case might represent more than one specific type. Necessary micro linguistic positioning aids included the indexicality of human language and communication, thematization rules, approaches of agency and positioning analysis ( Kruse, 2015 ; Lucius-Hoene and Deppermann, 2004 ), syntax (e.g. the use of active versus passive constructions) as well as the pragmatics (interaction and positioning) of linguistic actions.

Cases differed primarily by the extent of awareness development within the process. Below, the four different groups are characterized by their positioning within the typing model, the implementation of coaching contents (see Table 2 ) and their leadership concept (see Table 3 ).

**Type: Approachables on the sidelines** . In line with their position in the typing model, approachables on the sidelines exhibit little to no self-reflection or role reflection and low values on the axis “commitment shown in the implementation of coaching contents”. In contrast to the *realistic succeeders* , the approachables on the sidelines shows (no longer) any attempts to actively initiate change.

They describe their role as “ *team leader who, at times, uhm, appears at the sidelines* ” (Physical therapist). Their leadership concept is expressed by being approachable. Passive leadership is viewed favorably, “ *a good leader is someone, uh, where you can* ' *t tell that they* ' *re a leader* ” and “ *the more discreet, the better* ” (Physical therapist). Initially, absolute freedom of action is granted and an authoritarian leadership style is explicitly rejected. Leadership becomes necessary only in case of deficiencies and lack of work ethic and instructions are then issued.

In contrast to the group of realistic succeeders, they interpret the assumption of leadership responsibility as a lack of trust and a sign of general deficiencies. This type of leader is characterized by avoiding feedback and criticism, restrictive error management, intransparent decision-making and assessment criteria and poor decision-making ability. Further, this type desires a homogeneous team. It dichotomizes team members into “ *good fit* ” and “ *poor fit* ”, with team members who are a poor fit becoming apparent in conflict situations. These team leaders feel their center's framework conditions substantially restrict their options for action and they have accepted these restrictions. The reported lack of time is viewed as unalterable and rigid procedures guide these leaders. The *approachables on the sidelines* group is guided by rigid procedures and structures, deeming it unnecessary to alter processes: “ *That was (.)/the fact is, we have a procedure, our procedures are very clear//* ” (Nurse). They see no latitude for initiating changes and no need for team development measures. These team leaders particularly reject any additional work required to implement training contents. Passiveness is justified and legitimized by immutable framework conditions.

**Type: Distanced overseers** . The group of *distanced overseers* exhibits moderate values on both the reflection and commitment axes. They have excellent reflection skills regarding structures and processes, are very eloquent and often speak in metaphors:

But I have since I, uh, (.) yes, since I've become the leader, very deliberately taken the view that, uh (…) uhm, in a manner of speaking, a patient, particularly in a psychotherapeutic center, is a subject. And on the subject level (.) uhm, there is no hierarchically organized truth. That might be a little philosophical, but I believe you understand what I mean (…) (Physician)

However, *distanced overseers* do not extensively grapple with experiential processes regarding themselves individually or their work in the team. In line with their leadership concept, they view their style as more authoritative. These leaders exhibit pronounced positional self-esteem and situational authoritarian demeanor. This is expressed, for instance, by stating that they know their potential influence to “ *take advantage of the balance of power and, uh, strengthen positions, right?* ” (Physician). In the social realm, they establish hierarchy-based boundaries by clearly distinguishing between their personal role and professional self-concept: “ *Well, I* ' *m performing a function and am not in a personal role, right, that* ' *s very important to me* ” (Physician). Further characteristics are exemplary behavior, integrative work, call for the assumption of responsibility, fostering supervision and continued education, support of human resources development and the analysis of processes and structures. Training contents are endorsed, but rarely implemented. Unlike the group of *approachables on the sidelines* , these leaders do not justify the lack of implementation by time constraints, but rather by their deliberate identification with the idea that employees develop independently in a self-organizing system and that self-reflective processes will take place under any conditions.

**Type: Realistic succeeders** . *Realistic succeeders* are open and tolerant toward the views of all team members and represented professions. The following criteria characterize this group: reported implementation of the learned coaching contents, achievement of a reflected awareness development process and the protection of personal boundaries through an attitude of high personal responsibility in the leadership role. This group developed strategies that facilitated the implementation of training contents in the current context and said strategies can be viewed as success criteria for the implementation of coaching-based leadership interventions. Despite perceived economic pressures in the form of a constant sense of time pressure and resource shortage, *realistic succeeders* seek new ways to shape their work environment and change it where possible. Unlike *approachables on the sidelines* , who give up in light of this situation, *realistic succeeders* continually explore the current framework conditions and weigh their options.

Realistic succeeders depart from conventional frameworks to create new opportunities for encounters. As shown by the quote below, one physician took the initiative to look for an alternative space to conduct team development meetings, allowing for more creativity with the team and facilitating encounters: “ *We did that outside the center. We met at the creative arts therapists, uh, at her studio. That was a very different environment, which was very helpful.* ” (Physician).

Relationships of recognition and equality are actively fostered particularly in interactive situations within the team, where team leaders act as role models. This is expressed, for instance, in the form of worries about the various team members receiving equal speaking time: “[…] *So, in the professional discussion (.) everyone now gets the same amount of speaking time. So, the creative arts therapist gets just as much time as members (.) from the mindfulness area, from/relaxation training and so on* ” (Physician).

Team leaders practice this attitude and bring it to the forefront in the context of their leadership tasks. This includes being open and transparent about their weaknesses and errors, whether in the team by interactively setting an example in meetings, in one-on-one conversations with team members, or in patient contact: *(…)* “ *that you are transparent with the patient, too* *and* *say, we are all human* ” (Physician). These team leaders have confidence in their team's abilities, encourage team members to act independently and remain approachable for support. They achieve a balance between support and autonomy:

But to get the balance right (.), uh, support, help, but also (…), how can I say this, but also, well, that/you should be able to do that on your own now, or try doing it alone. And if it doesn’t work, I'll still be there (…) (Psychological psychotherapist)

This contrasts with *approachables on the sidelines* , who dichotomize team members into “poor fit” and “good fit.” When problems arise, *approachables on the sidelines* largely place the blame on individual persons: “ *It* ' *s also always due to the person* ” (Nurse) and prioritize the use of subjective bases of evaluation: “ *And the people were odd, too* *and* *then, err (.) I fired them all (.)// (laughing) and hired new people* ” (Nurse).

An essential element in terms of adherence to priorities and structure and the team leaders' understanding of their role was the insight that, in line with a grassroots democratic approach for the team's benefit, not all team members would be satisfied with the decisions made. This attitude is evidenced by the following quote: “ *We can* ' *t always (.) satisfy everyone. It has now/We have now heard everything, we thought about everything together* *and* *now we have/a decision has to be made.* ” (Physician).

They differ from the group of *dedicated sensitives* , who strongly desire harmony and wish to “ *become one* ” with the team. Dedicated sensitives will tend to refuse to discuss negative or onerous topics with team members: “ *I don’t mind holding performance reviews so much, but well, (* … *) what if it, well, (* … *) if difficult things come up.* ” (Occupational therapist).

**Type: Dedicated sensitives** . *Dedicated sensitives* exhibit the highest values on the axes of commitment as well as observed reflection: They are characterized by pronounced self-reflection and critical self-examination and strive for self-optimization and continued development. Simultaneously, they see themselves as lacking authority in their leadership role: “ *In my case, I* ' *m noticing what I* ' *m missing now (laughs) (…) or what I think I* ' *m missing. Well, just the more authoritarian stuff* ” (Occupational therapist). In contrast to the group of *distanced overseers* , members of this group see themselves as very approachable in the social realm: Interpersonal and emotional phenomena are deemed particularly important. They discuss fostering harmonious relationships and sensitivity to emotions and moods that can influence team processes: “ *Uh, (* … *) it* ' *s important to pay attention to atmospheres, too* *and* *maybe to probe sometimes, to be highly sensitive* ” (Occupational therapist). In dealing with structures and limited resources, priorities are set with the goal of “ *being able to take time to have a lot of conversations* ”. Important leadership guidelines are honesty, fostering openness and considering differences due to age and experience. There is a desire for mutual appreciation and goodwill and for growing together with the team (“ *becoming one* ”). Regarding the implementation of coaching contents, this leadership type is highly motivated and personally committed to implementing them. Differences to the *realistic succeeders* are particularly pronounced concerning personal responsibility: The personal role blurs with the professional role and this is coupled with pronounced perfectionism, insecurities and self-criticism.

## Discussion

The use of the TLCP proved to be a promising intervention for initiating and enhancing an awareness development process in team leaders. It particularly benefited team leaders who experienced substantial development of awareness as shown by their positioning in the core category concerning reflection on their position as team leaders and their leadership role within their teams and who were simultaneously able to implement coaching contents in their daily routine. According to the typing model, this corresponds to the realistic succeeders and dedicated sensitives.

Since attitudes are believed to be revealed in social interactions rather than being accessible to individuals through reflection, conclusions about the underlying mechanisms can be drawn with the aid of the reconstructive analysis of the reported information ( Breuer *et al.* , 2017 ). High awareness development and reflection on the leadership role were found to be prerequisites for the implementation of the learned content, coupled with the ability to generate more room for maneuver through creative solutions and persistence. No systematic relationship was found between the identified types on the one hand and professions on the other. Therefore, profession does not appear to influence the potential adoption and implementation of the coaching tool. Rather, the degree of openness to different perspectives and the wish to continuously shape the work environment appear to be decisive criteria for adopting the TLCP.

With regard to the context in which the TLCP was implemented, high expectations for medical care and a simultaneous lack of personal and financial resources were reported, concurring with the German Medical Association ( Bundesärztekammer, 2007 ). Lack of time for team development and leadership were particularly highlighted. The results show that the group of realistic succeeders plays a special role in terms of their handling of this lack of resources. Team leaders who managed to oscillate between acceptance of existing conditions and change within the given scope of action created the necessary conditions for implementing team coaching at their centers. The qualitative data show that interdisciplinary collaboration is especially important in the rehabilitation context but also places great demands on team leaders. Realistic succeeders managed to strengthen the sense of community in the team. This is consistent with studies according to which team identity moderates the relationship between diversity and effectiveness. This suggests that commitment and attraction to their team enhances team members' ability to cooperate ( Mitchell *et al.* , 2011 ). Another strategy of successful team leaders involves generating openness norms in the interprofessional team. This underscore results from a health services research study showing that the degree of openness, that is, the willingness to openly take different perspectives into consideration and question one's own position, was decisive for the ability to overcome professional barriers, such as the perceived threat to professional identity posed by innovation ( Mitchell and Boyle, 2015 ). To facilitate change in role effectiveness, team leaders' attitude appears to be a decisive parameter for change and is associated with applied strategies and behaviors. In the study, this attitude is evidenced by high reflectiveness of team leaders and their willingness to continue developing in their leadership role. It has been demonstrated that leaders who value and request feedback for themselves are perceived as more effective in their coaching role ( Steelman and Wolfeld, 2018 ).

Further the active creation of relationships of recognition, the recognition of and transparency regarding their strengths and weaknesses as the basis for leading different professions as colleagues, was a promising strategy. This is consistent with approaches of shared leadership, which aim to focus on the leadership and coaching of colleagues. The listed strategies also relate to research results on the construct of humility: Leadership humility, based on three dimensions of humility, can affect team performance by promoting constructive interpersonal processes ( Owens and Hekman, 2016 ; Owens *et al.* , 2011 , 2013 ). Extrapolated to an interprofessional team, the theory states that each profession can recognize its strengths and weaknesses and thereby clarify its role (reflected awareness). Simultaneously the strengths and weaknesses of the other professions are appreciated (interpersonal awareness) and openness toward new perspectives and information (executive function) is developed.

Unlike the other types in the model, the group of realistic succeeders managed to integrate self-care as a critical element of leadership. Since leaders who practice self-care are believed to minimize their risk of burnout ( Ghossoub *et al.* , 2020 ), this aspect appears increasingly relevant, including given the figures mentioned above from the German Medical Association ( Bundesärztekammer, 2007 ) and should be taken into consideration when developing future training programs.

A phenomenon reported by all interviewees was a perceived lack of time. Nevertheless, realistic succeeders were able to create time despite reporting time pressure. The reports of a perceived lack of time for performing leadership duties should be considered and lead to the initiation of structural changes to enable team leaders to perform management responsibilities and thereby ensure optimal patient care.

Methodologically, the contextual knowledge of the trainers was used as a data source, operationalized through the evaluation in the analysis group, with the goal of increasing sensitivity in theory formation and variability ( Strauss, 1991 ). This was additionally implemented by questioning presuppositions, deliberately arguing opposite positions and reflecting on own attitudes and impressions. The typing model developed in the primary analysis did not permit typing all cases since there was an excess of characteristics. This indicates that the developed theory is neither too specific or general ( Breuer *et al.* , 2017 ). The developed typology is intended as a system that provides orientation for determining potential factors influencing the success or failure of the intervention. In cases that did not fit, the reasons for exclusion were discussed and recorded in the analysis group. In developing the described structure, care was taken to consider the conflict between the level of detail of the individual cases and the level of abstraction required for theory development and reflect on it in the analysis group. Technically, it might be argued that the typing model is confirmed by the absence of a type with maximum development of awareness and simultaneously low commitment. Hypothetically, lack of commitment despite increasing understanding of one's role could give rise to the phenomenon of cognitive dissonance. This phenomenon arises when one's behavior and attitude are perceived as inconsistent, causing internal dissonance ( Festinger, 1962 ). The desire to resolve this dissonance makes it unlikely for individuals to remain in this state permanently.

Nevertheless, due to the varied influencing factors and lack of control, no transparent cause-effect relationships can be demonstrated using the present design. Described causal relationships are based on the team leaders' narratives in the interviews.

## Conclusion

Team leaders were surveyed on their experience of a coaching process they implemented in their teams. The interviews were analyzed using the grounded theory method (GTM) and resulted in a typing model, describing four types of leaders (Approachables on the sidelines, Distanced overseers, Realistic succeeders and Dedicated sensitives), who differed in terms of developing awareness. Those team leaders who were able to use the TLCP to initiate and reinforce an awareness development process related to reflecting on their position and leadership role were also more likely to report on implementing the trained coaching content. For future training, leadership concepts closely related to reflection on one's own leadership role as well as interventions for strengthening relationships of recognition should be considered. One objective in leading interprofessional teams should be the generation of openness norms, which allow each team member to humbly recognize his or her own strengths and weaknesses, obtain clarity about his or her own role, learn to appreciate the strengths and weaknesses of other team members and develop openness toward new perspectives and information. Using methods and their practiced behavior, leaders can become role models for interprofessional collaboration and exert influence to reduce the negative impact of social categorization and take advantage of interprofessional collaboration in terms of the resulting synergistic effects.

## Figures and Tables

**Table 1:** Developing the analytical core category “Development of Awareness”

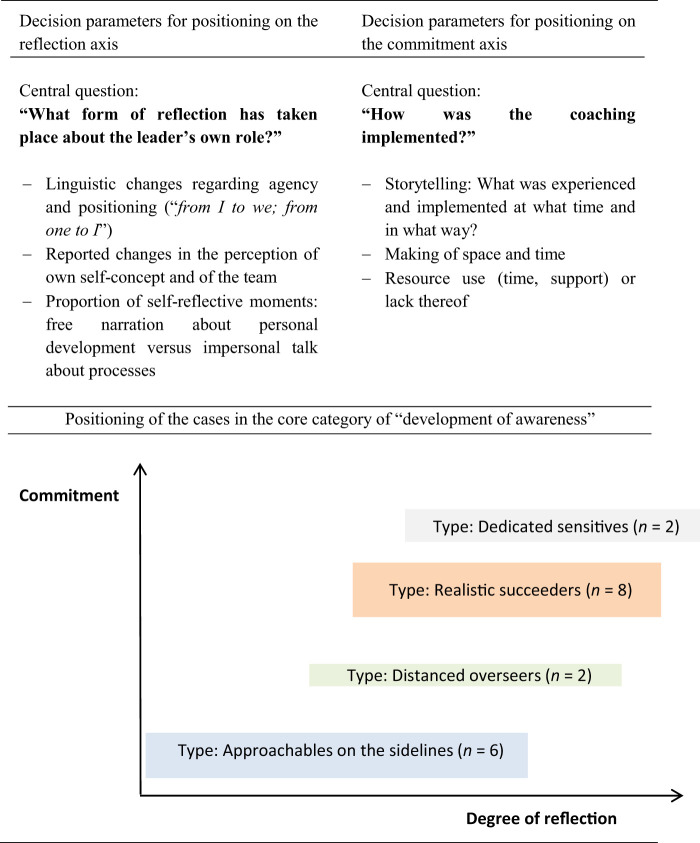

**Table 2:** Characteristics of the four types regarding self-reflection and implementation of coaching contents

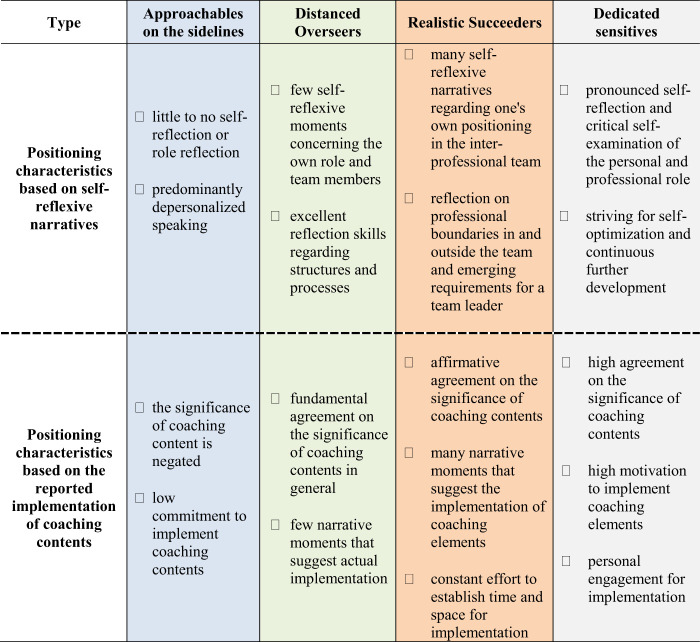

**Table 3:** Characteristics of the four types regarding their leadership concept